# Abdominoplasty today

**Published:** 2008-10

**Authors:** Dinesh Bhargava

**Affiliations:** Director, ‘A New Image”, Center for Aesthetic Plastic Surgery, Head of Department of Aesthetic and Plastic Surgery

While the changes of the abdomen that occur after pregnancy are as old as the history of the human race our addressing of these concerns has only been since the last century when Kelly[1] first did a panniculus excision through a transverse incision primarily as a debulking procedure. Since then the evolution in our understanding of the microcirculation, the nuances of lymphatic drainage and the pathophysiology of the deformity have helped us create a more aesthetic end result from our surgical endeavours.

## HISTORY

Our early attempts to correct the abdominal deformity were directed to surgical excision with little attempt to concern ourselves with the lay of the scar or the aesthetic details. This was the primitive era of debulking of the excess skin and the subcutaneous tissue. This therefore confined our patient selection to the extreme situations. At that time the other issues of safety of anaesthesia and infection made any surgical intervention or ‘only if you must’ category. The need to re-establish the abdominal land marks after this form of debulking was introduced by Thorek when he introduced the procedure to preserve the umbilicus.

As our understanding of the general surgical principles grew, as we learned to control the issues related to infections and risks related to anaesthesia were addressed and reduced to acceptable levels, we started to look at this procedure with a point of view of aesthetics. Pitanguy[2] was amongst the early surgeons who addressed the issue of the placement of the incisions in the lower abdomen in a ‘W’ manner. Reganult[3] and Grazer[4] advocated placing the incision in the ‘bikini line’. The decades between 60- 80 saw the development of the abdominoplasty as a procedure with a presumption of the vertical excess of skin and laxity of the abdominal musculature –a natural outcome of pregnancies. The variation being in the design of the incision, bicycle-handlebar incision by Baroudi and Moraes[5], the plication of the abdominal muscular unit (linea alba: Calia,[6] the external oblique: Psillakis[7]) and the extent of dissection based on patient's individual needs.

The introduction of liposuction[8] in the early 1980s changed for ever our perception of abdominoplasty. While excision of the excess tissue was still the primary objective the contouring of the tissues left behind became an issue to consider as well. Liposuction became a part of the excisional surgery. Our initial encounters with complications such as poor healing if not dehiscence of incision and seroma formation made for the serious consideration of the risks of the combination. During this time we saw a definite emergence of consideration of aesthetics of the abdomen in this excisional surgery.

I do believe that the next water shed development came in 1995 with publication by Dr. Ted Lockwood[9] who developed the procedure he called Lateral Tension Abdominoplasty as an outcome of the lower body lift procedures he was doing in the bariatric surgery patients.

## LATERAL TENSION ABDOMINOPLASTY

In his publication in Plastic and Reconstructive Surgery in 1995, an article worth reading for any student of Plastic Surgery, he asserted that abdominoplasty was not a two dimension issue but a three dimensional procedure.

Dr. Lockwood's operation, the lateral tension abdominoplasty, was not just a variation on the old theme but a new concept in the pathophysiology of the abdominal laxity and its management.

His assertions were

1. In the post partum abdomen the excess of skin is not just vertical, but circumferential.
2. The lax skin tends to move medially and caudally when in an examining position (standing) which had given rise to the concept of central abdominal excess as being only in the mid line in the classic abdominoplasty.
3. Muscle correction is an integral part of creating a flat abdomen.
4. A continuous dissection in the subcutaneous tissue is not necessary to mobilize tissue for excision.
5. Discontinuous dissection is done to mobilize tissue
6. Use of liposuction to contour the torso, tissues are left behind, not excised.

With these assumptions he devised the surgery that was different from the classic in terms of the placement of scar, the extent and nature of dissection, the direction of the tension and pull and the recognition of the tension bearing layer he called the superficial facial system (the SFS).

The evolution of this thought process was to meet the demands of people who were not only looking for a relief of a burden of weight but seeking an aesthetically attractive abdomen as an end result [Drawing 1].

**Drawing 1:**
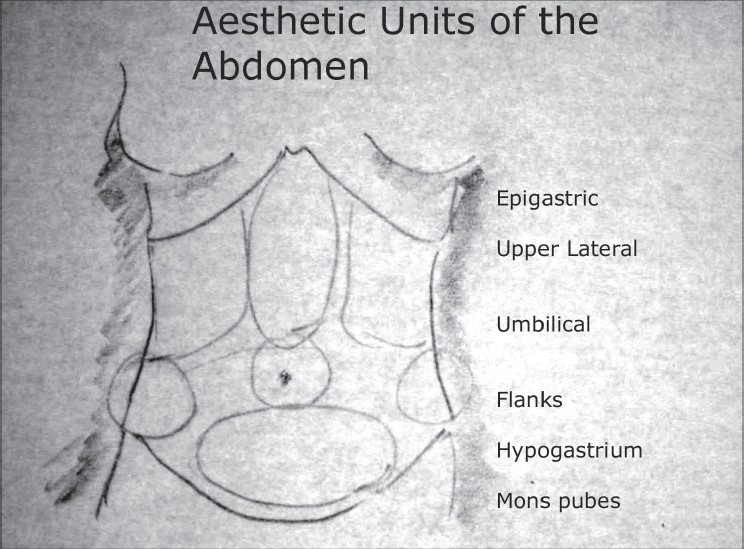
Aesthetic Units of the abdomen

### Elements of aesthetics in abdominal contouring

1. Tight and firm anterior abdominal skin, preferably without stretch marks and visible scars.
2. Good muscle tone, flat abdomen
3. Contours to show paucity of subcutaneous tissue including a central depression in the epigastrium.
4. The ‘six pack’ appearance.
5. Umbilicus without the upper hooding
6. Youthful lower abdomen and the mons.
7. Frontal silhouette to show a continuous sinuous shape at the hip region.

With the these assumptions and the objectives in clear view he formulated a general plan that included the following

1. Preoperative markings optimize the placement of the scar with a view to excise the excess tissue from both central and lateral abdomen.
2. Liposuction of the upper lateral abdomen, the central epigastric region, the hip roll and the flanks. This was the element of discontinuous dissection.
3. Continuous dissection of the lower abdominal flap limited to the excision lines.
4. Limited dissection to expose the central abdomen for repair of the diastasis of muscles.
5. Correction of the diastasis of linea alba.
6. Correction of the ageing changes of the mons pubis
7. Closure with the tension directed laterally using the SFS layer as a major support for closure.
8. Relocation of the umbilicus.

The lateral tension abdominoplasty was thus introduced to us. Since then and over 12 years or so I have done this operation keeping these principles as guide posts. Several innovations[10–12] have been added to the original description of the technique to improve on the results and to incorporate newer technology and concepts. I will attempt to go over how I have come to do this operation and share with the reader as to the reasoning for my decisions.

### Selection of patients, indications of surgery

Laxity of the abdomen musculature and skin are the issues this operation addresses. Other deformities such as lipodystrophy and silhouette deformities could also be addressed by this procedure. Reason for this surgery, however, is the patient's desire for the correction and her/his willingness to accept the risks and responsibilities that come with the surgery.

Age, Diabetes mellitus, hypertension, cardiac conditions, upper abdominal scars and excess weight are all relative contraindications. A history of DVT, bleeding diathesis, and morbid obesity would be considered absolute contraindications. Abdominoplasty is best done after the family is complete and no further pregnancy is expected. While pregnancy post abdominoplasty is possible, it is not advisable since it will undo the effort put in.

Smoking[13] is a concern. It affects the microcirculation and thereby affects the post operative healing, apart from the pulmonary effects that it has. Cessation of smoking at least 2 weeks prior to the procedure is considered by many as a mandatory requirement for surgery. This is not only to improve the pulmonary function but also to measure the patient's desire and commitment for the procedure.

In bariatric patients abdominoplasty may be a sole procedure but is often a part correction of other regions as well. These patients form a separate subset. The indications and risks are different as well.

A healthy person or one with medical concerns that are well controlled will help with the care and out come.

### Planning [Drawing 2]

**Drawing 2:**
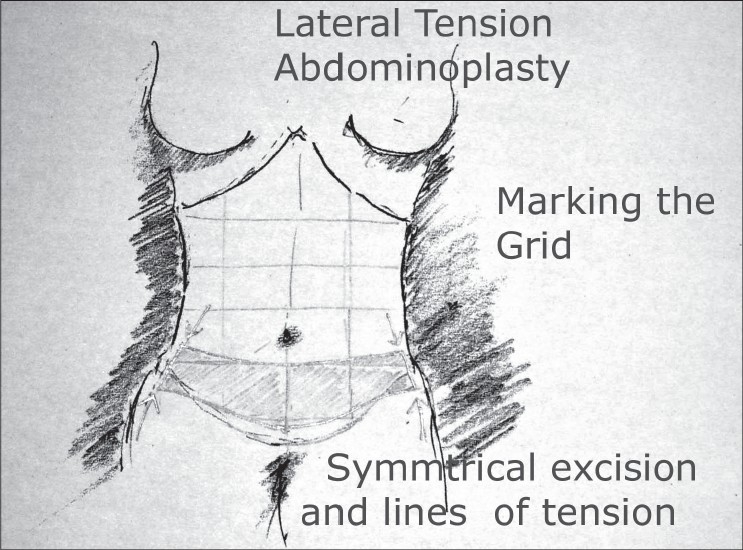
Marking of a grid for symmetrical excision and scar placement

Preoperative evaluation of the abdomen with attention to the site of fat deposits, excess of skin on the lateral region and the anterior midline, the divarication of recti, any hernias or scars of previous surgery all need to be noted.

Identify the anatomical landmarks and make a grid[14] on the abdominal wall. Use of grid using a level may help with the grid pattern. This helps with symmetrical placement of the incision and a subsequent incision closure. Mark the midline, the linea alba and semilunaris, the anterior superior iliac spine, the xiphoid process and the costal margins.

The regions of liposuction are noted and marked including areas of the upper abdominal region both lateral and epigastric region. The flanks, the hips and the mons regions are marked as well. The sites of incisions are marked [Drawing 3,4].

**Drawing 3:**
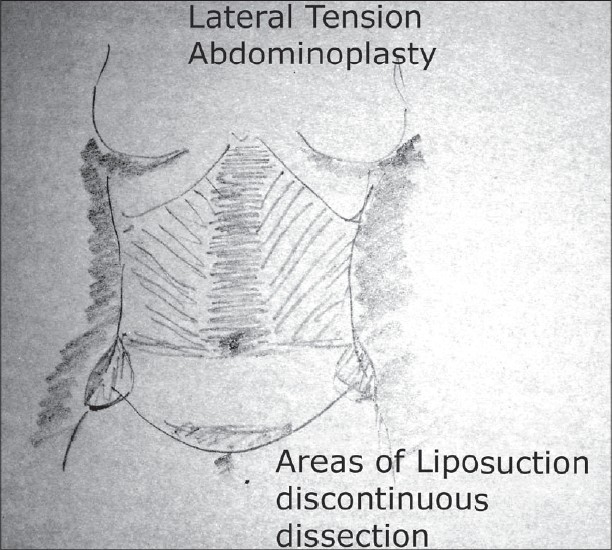
Areas of discontinuous dissection, liposuction

**Drawing 4:**
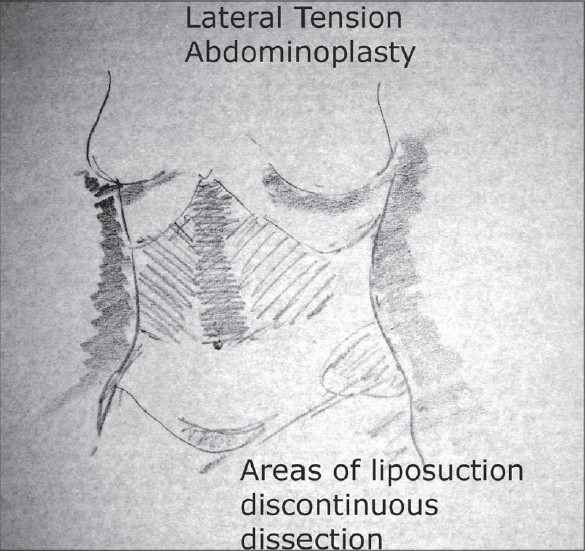
Areas of discontinuous dissection, liposuction, oblique view

With the help of the patient identify the preferable region for the placement of the final incision. Let the patient wear the garment of her choice to determine the extent of the safe region. Within this region mark where you anticipate the final incision will be. The marking of the incisions is then done recognizing the direction of the ‘pull’ that will be required to, not only excise, but also maximize the aesthetic out come. In this regard the pubes region will be raised as the abdominal skin is brought down, the hip and flank region will be raised as well, as the lateral tension closure is accomplished. The areas for liposuction (discontinuous dissection) are marked as well, as are the regions for continuous dissection. A symmetrical excision on the two sides is judged before the patient is anesthetized.

### Anaesthesia

General anaesthesia is the common option while regional anaesthesia remains a choice. The decision is best arrived by a consultation between the patient, the anaesthesiologist and the surgeon. The need for muscle relaxation during the repair of the diastasis should be discussed.

### Items of concern

1. DVT prevention needs to addressed with the use of sequential compression devices.
2. A urinary catheter may be deemed necessary as well.
3. Body temperature during the surgery. Use of body warmers is recommended.

## SURGERY

### Liposuction

Liposuction, whether tumescent[15] or Ultra sonic is used. This is primarily to provide for a discontinuous dissection but it also helps contour the areas so treated. The contouring of the epigastric region, the linea alba, the flanks and the upper lateral abdomen are so treated to the extent of preoperative planning. The mons lipodystrophy is treated at this time as well.

Ultrasound Assisted Liposuction[16] (UAL) offers a significant advantage in the sense it preserves the vasculature to the flaps better than the other modalities that we know. It also produces less bleeding and subsequent swelling in the region treated. While there are concerns of the burn at entry site with this modality, placing the incision in the lower abdomen obviates any problem since this part of the abdominal skin will be excised.

### Incision and exposure [Drawing 5]

**Drawing 5:**
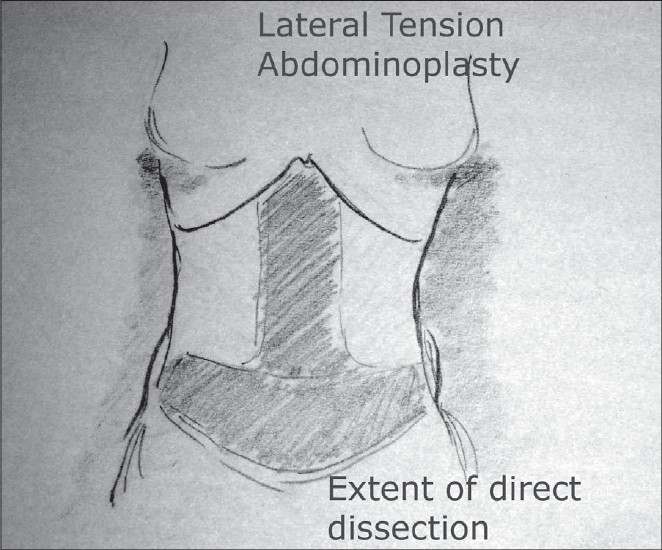
Extent of direct dissection

The central lower abdominal incision is made first. This is deepened through the Scarpa's fascia to the level just above the muscle. The superficial inferior epigastric vessels are identified, clamped and ligated. The dissection at this level is easy and nearly bloodless. There is a school of thought which states that a layer of fat left behind on the muscle provides for a better lymphatic drainage from the operative site and therefore helps reduce seroma formation. I have found the dissection in between the layers of fat more tedious and messy especially after liposuction and since I have had very little problems with the seroma, I have felt that this extra exercise is not clinically significant. The dissection is carried to the level of the excision line on the abdominal flap. The perforators need to be identified, clamped and ligated. The incision around the umbilicus is made and the stalk delivered into the operative site. The dissection is continued in the mid line up to the xiphoid region identifying the medial border of the rectus sheath and the divarication. This is often no more than a 6 to 7.5 cm. wide tunnel.

### Repair

The divarication of the recti is then repaired using a non absorbable suture. I have used TevDeck, Surgidac and Prolene, but find a braided suture better to tie and hold. This repair is done in two layers, the first being interrupted and the second layer of continuous running sutures. This is often enough in terms of abdominal tightening, but occasionally lateral sutures in the external oblique may be deemed necessary. One must, however, be cognizant of creating intraabdominal hypertension.[17] Intraabdominal hypertension can contribute to venous compression.

### Planning excision [Drawing 6]

**Drawing 6:**
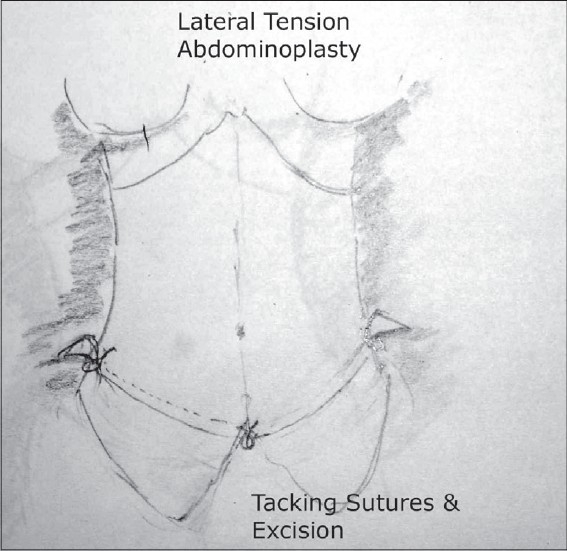
Tacking sutures and excision

At this time the patient is placed in a flexed position and an incision made at right angle at the extreme lateral end of the lower abdominal incision. This marks the line of tension of the lateral pull. Tacking sutures are placed at the two lateral ends and at the mid line. The sutures at the lateral end are under high tension and would move the hip region up and medially as they would pull the abdomen laterally and downwards. The mid line tacking sutures are expected to be under less tension. This will give a pattern of excision which is done without any central mid line tension.

### Excision

The demarcated segment of tissue is excised with more excision being done in the deeper layers than in the skin. The location of the umbilical stalk is noted on the anterior abdominal wall. Hemostasis is assured both at the line of excision and under the flap. Lateral ‘dog ear’ correction needs to be done to set the incision at an appropriate level as judged in the preoperative planning.

### Closure [Drawing 7]

**Drawing 7:**
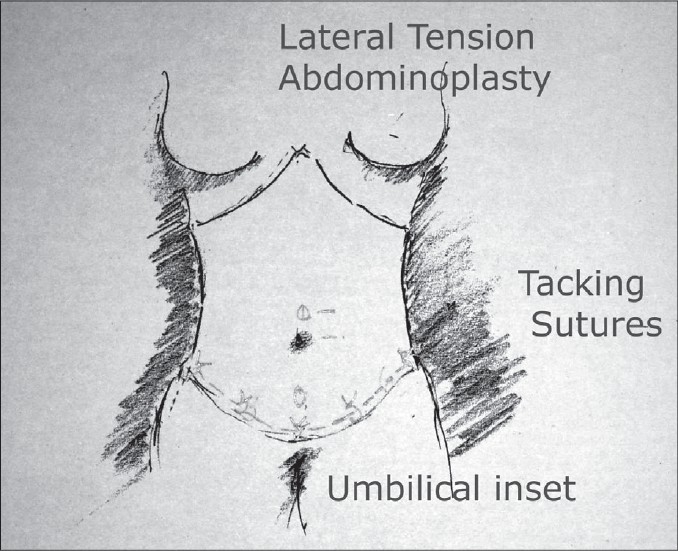
Umbilical inset

This is the crucial issue in this surgery. A layer of interrupted non absorbable sutures are placed in the SFS layer to secure the closure. This is done under significant tension laterally tightening the anterior abdominal wall and pulling up the flank and the hip region. This is responsible for the improvement of the frontal silhouette.

### Inset of the umbilicus[18] [Drawing 8]

**Drawing 8:**
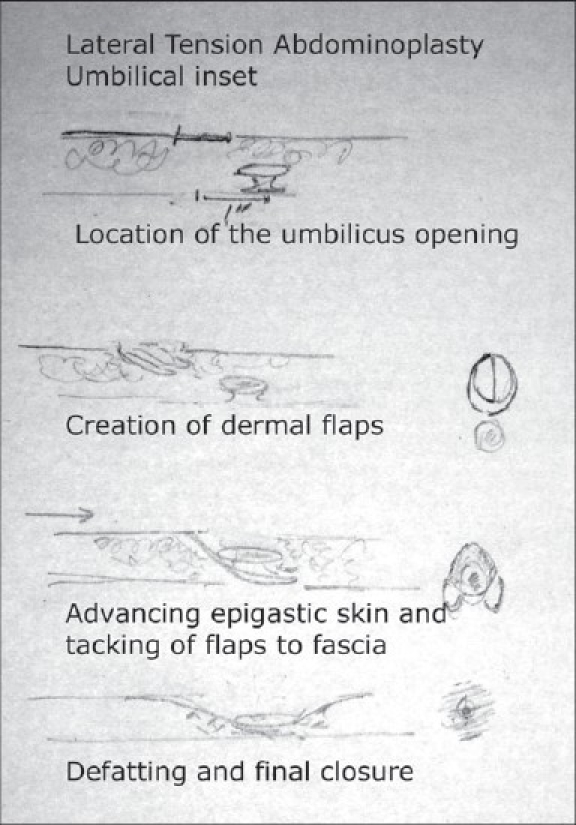
Technique of umbilical inset

An incision is made 2 cm above where the umbilical position was marked. A circular opening is designed. The skin is de-epithelialised and two flaps are created. The periumbilical region is defatted to create a more aesthetic umbilical region. The two dermal flaps are then tagged down at the level of the umbilicus to the deep fascia to tighten the supraumbilical region of the abdominal skin and helping take the tension off from the central lower abdominal closure. The closure of the umbilical incision is done layers.

### Final closure [Drawing 9]

**Drawing 9:**
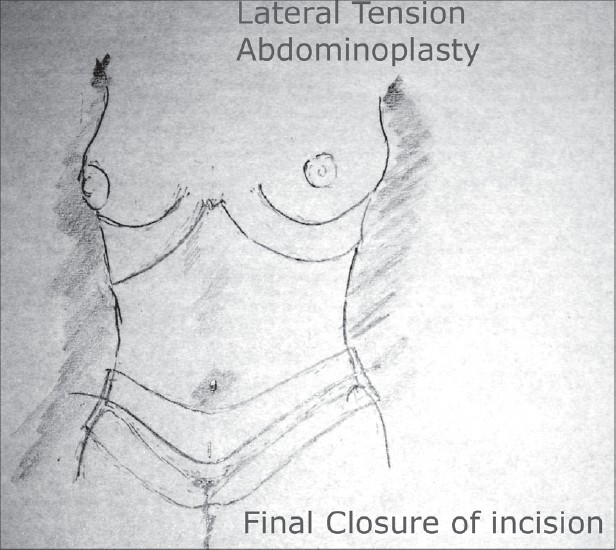
Final closure of incision

The closure of the abdominal region continues with the placement of a flat silicone suction drain in the mid line and the lower abdomen. The second closure is dermal layer and the last layer is that of the subcuticular layer with an absorbable monofilament suture. Often the original umbilical site incision needs to be closed in the lower abdomen. Bringing the subcutaneous adipose tissue together will allow for a flat incision. This vertical incision heals well and in my opinion is worth the advantages (see below) of the lateral tension abdominoplasty.

### Dressing

A non adhesive dressing much like a Vaseline gauze dressing and some absorbable gauze is used. Only mild compression for comfort is necessary since we have a suction drain in place.

### Post operative care

The patient is encouraged to ambulate with 12 - 18 hours. During this period pain management is critical. The drains can be removed with in 24 hours. Sutures are removed by the 5-6^th^ day. The compression garments are kept for 6 weeks for the resolution of swelling and for support.

**Figure 1:**
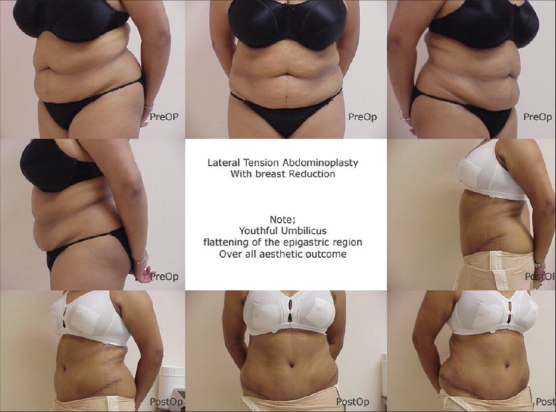
LTA for laxity and lipodystrophy, 42 yr female. Note youthful umbilical region and flattening of epigastric region

Figure 1–Figure 3 are some examples.

**Figure 2:**
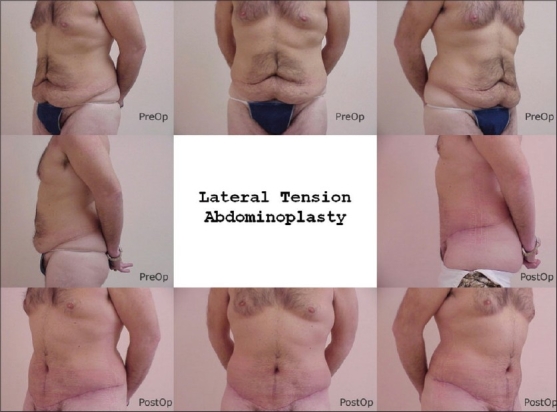
LTA for laxity after weight loss, 47 yr male. Note over all aesthetic presentation

**Figure 3:**
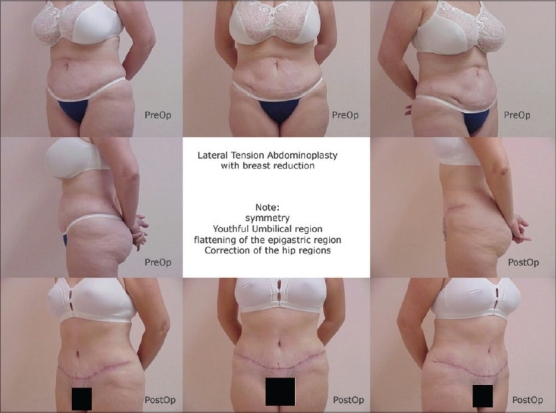
LTA for laxity and post C Section, 43 yr, female. Note youthful umbilical region and flattening of epigastric region

### Advantages

1. Addressing all the aesthetic segments of the abdomen results in a more natural outcome.
2. Robust skin flap, minimal risk of ischemic changes in the flap.
3. Location of the scar is stable and consistent.
4. Ambulation earlier and with less limitations.
5. Redefining the hip contours.
6. Correction of the supraumbilical laxity of the abdominal skin.
7. Versatility of the procedure. With some variations in design, the procedure can be adapted to a variety of abdominal laxity deformities.

### Concerns

1. Time: 3.5 to 4 hours longer than the standard abdominoplasty
2. Incisions are longer. Not always possible to excise the umbilical region and hence a vertical scar is often present in the lower abdomen.
3. The lower abdominal scar can often be hidden but may not be possible with the low lying pants of the modern generation

### Complications

These can be grouped under those that are related to anaesthesia and those related to surgery.

Complications related to anaesthesia: These are obviously related to the kind of anaesthesia used and due deliberation regarding these risks must be done by the anaesthesiologist and the patient. A healthy person and/or one who has been medically optimized offers us the best opportunity to reduce these risks. The concerns about drug reaction and allergies remain a concern despite a good history and care.

In addition the body temperature control and monitoring need to be addressed. The use of sequential compression device during the surgery to prevent DVT have already been mentioned.

### Complications related to surgery:[19,20]

**Table:** 

1. Haematoma and / or seroma formation	10.6%
2. Unacceptable scars (placement, wide and asymmetrical scars, prolonged redness, unnatural looking umbilicus)	7.9%
3. Infection	5.6%
4. Tissue loss	5.6%
5. Bleeding	0.4%
6. Intra abdominal hypertension	rare
7. DVT and pulmonary embolus	0.28%

Many of these can be kept at bay by proper planning and accurate execution of the procedure planned. Despite the best efforts, complications will occur. The outcome can still be controlled with early recognition and steps taken promptly to remedy the concerns. It is imperative for the surgeon to provide a close postoperative follow up, since tough decisions may be required in this critical time frame which will affect the long term results.

From its inception and publication in the mid 90's the Lateral Tension Abdominoplasty has evolved. Its versatility, reliability and overall satisfaction of the outcome makes this procedure worthy of consideration when considering correction of abdominal laxity.
